# The Prevalence of Long-Term Lower Urinary Tract Symptoms Among Women Who Previously Experienced Postpartum Urinary Retention—A Cross Sectional Study

**DOI:** 10.3390/jcm14124184

**Published:** 2025-06-12

**Authors:** Yoav Baruch, Marta Barba, Tomaso Melocchi, Alice Cola, Alessandra Inzoli, Matteo Frigerio

**Affiliations:** 1 Urogynecology and Pelvic Floor Unit, Department of Obstetrics and Gynecology, Tel Aviv Medical Center, Tel Aviv University, Tel Aviv-Yafo 6997801, Israel; 2 Department of Obstetrics and Gynecology, ASST Monza, San Gerardo Hospital, University Milano-Bicocca, 20900 Monza, Italy; m.barba8792@gmail.com (M.B.); t.melocchi@campus.unimib.it (T.M.); alice.cola1@gmail.com (A.C.); a.inzoloi2@campus.unimib.it (A.I.); frigerio86@gmail.com (M.F.)

**Keywords:** PPUR, postpartum urinary retention, voiding dysfunction, risk factors, LUTS

## Abstract

**Background**: Postpartum urinary retention (PPUR) typically resolves within the first three days following delivery. However, in rare instances, it may persist beyond 72 h and, in some cases, extend for several weeks. The current study aimed to evaluate long-term sequelae in women who endured PPUR following vaginal delivery. **Methods**: Between January 2013 and December 2019, 362 women who experienced PPUR following delivery at our institution were identified and subsequently invited to complete the UDI-6 questionnaire that serves to assess lower urinary tract symptoms. The questionnaires were filled out and returned by 242 women (66.8%). **Results**: Participants who had no urinary complaints (145/242; 60%) were assigned to Group 1. Of the 97 women allocated to Group 2 (97/242; 40%), 96 reported only mild urinary symptoms, while just 1 individual scored above the threshold of 33.3, suggesting elevated urinary distress. Risk factors known to be associated with PPUR were equally distributed among the two groups. A predominance of Caucasians was noted in Group 2 (*p* = 0.012). Voiding dysfunction (question 5 of UDI-6), taken separately, was proclaimed by 15 women from Group 2 (15/97 = 15.5%). When these were compared to the rest of the cohort (*n* = 227), an association with hypothyroidism was recognized (*p* = 0.036). Well-established risk factors for PPUR, such as nulliparity and epidural analgesia, were observed less frequently among women with persistent voiding dysfunction (*p* = 0.045 and *p* = 0.049, respectively), while postpartum uterine atony was more frequent (*p* = 0.047). Significant long-term effects after PPUR are uncommon. **Conclusions**: Hypothyroidism and postpartum uterine atony emerge as risk factors allied to long-term voiding dysfunction.

## 1. Introduction

Postpartum urinary retention (PPUR) is a postpartum manifestation with a reported incidence that varies widely subject to inconsistent definitions and the time interval of follow-up. Three clinical forms of PPUR have been described: overt, covert, and persistent. The overt form is defined by an inability to void within six hours of delivery or six hours after the removal of the urinary catheter, whereas covert retention refers to incomplete bladder emptying and consequently to enlarged post-voiding residual volume (PVRV) greater than 150 mL. Persistent urinary retention persists for more than three days and can linger for several weeks [1,2].

The reported incidence of PPUR varies widely. Systematic meta-analyses designed to estimate the overall prevalence of PPUR in women after vaginal delivery assert that the overall prevalence of overt and covert urinary retention approximates 1 to 2% and 11 to 13%, respectively [3,4,5]. A recent analysis of 18.289 parturients in Italy provided an overall overt PPUR rate of 1.4% [6]. Most PPUR cases require catheterization, either intermittent or continuous, and resolve within 72 h. The pathophysiology of PPUR has been linked to multiple factors, either physiological, neurological, or mechanical. Reported risk factors include age < 35 years, nulliparity, advanced gestational age (>41 weeks), epidural or systemic analgesia, premature rupture of membranes, labor induction, prolonged labor, Kristeller’s maneuver, meconium-stained amniotic fluid, episiotomy, instrumental delivery, perineal laceration, singleton pregnancy, and increased neonatal weight [2,4,6,7,8,9,10,11,12,13,14,15,16,17]. Minor independent risk factors linked to PPUR recently reported were non-Caucasian ethnicity and BMI of less than 30 kg/m^2^ at the end of the pregnancy [6]. Absence of spontaneous voiding before leaving the delivery room is another independent risk factor allied to PPUR [18]. Patients with overt PPUR who are unable to void spontaneously are easily detected, whilst those with covert PPUR who can void spontaneously but have a PVRV of >150 mL may remain unrecognized unless screened for residual volume. As abnormal PVRV is self-limited, the correct prevalence of covert PPUR is probably underestimated [16,19]. Taken together, the prognosis of PPUR is reportedly good, and PPUR is not expected to deter urinary function [12,19,20,21]. However, PPUR may be linked to long-term urogenital tract morbidity, and the lack of available data on its long-term consequences may deter prompt diagnosis and appropriate management. Persistent urinary retention can result in chronic voiding difficulties, bladder over-distension, perineal neuropathy, and irreversible detrusor damage, and in extreme cases may lead to bladder rupture [22]. The need to better define the magnitude of long-term lower urinary tract symptoms (LUTS) after PPUR remains indispensable. Because the literature provides only scant data, the true incidence and determinants of these sequelae remain unclear. Evidence supporting the notion that PPUR is harmless is lacking. Therefore, the aim of this study was to use the validated UDI-6 questionnaire to determine whether women who experienced PPUR after vaginal delivery report persistent lower urinary tract symptoms, and to identify obstetric or medical determinants that predispose them to prolonged voiding distress.

## 2. Materials and Methods

This was a single-center cross-sectional study of women diagnosed with PPUR at our tertiary university-affiliated medical center at Fondazione IRCCS San Gerardo dei Tintori, Monza, Italy, conducted over a seven-year period, from January 2013 to December 2019. Women (*n* = 362) identified by the diagnostic code were contacted by phone or mail and asked if they were willing to complete the UDI-6 questionnaire, a validated, self-administered 6-item questionnaire, widely used to assess symptoms associated with lower urinary tract dysfunction [23]. Informed consent was provided to be signed and returned. The questionnaires were e-mailed and returned between January and February 2023 (follow-up period of 3 to 10 years). Women who did not respond were not re-addressed. Altogether, 242 women completed the UDI-6 questionnaire. Inclusion criteria: singleton pregnancies delivered vaginally after 34 weeks of gestation and diagnosed with PPUR. The research protocol was approved by the local Institutional Review Board (Protocol Code ITA-PFDI-20). Clinical data were retrieved from a dedicated database software system used for antenatal care and assessment. Variables analyzed included population characteristics (age, ethnicity), medical and obstetric characteristics (comorbidities, autoimmune disorders, hypothyroidism, parity, GDM, hypertension, previous cesarean section, BMI at the end of the pregnancy, gestational age, polyhydramnios), intrapartum variables (analgesia type, oxytocin use, color of amniotic fluid, induction of labor, fetal presentation, mode of delivery, length of pushing phase, maternal position during delivery, blood loss, need for a manual placental removal, uterine atony, perineal injury, fever), and neonatal parameters (gender, OFC, length, weight) (depicted in Table 1). According to our policy, oxytocin is given subject to specific clinical indications. Epidural analgesia is approved upon women’s request at the anesthetist’s discretion. Instrumental birth is completed by vacuum extraction (Kiwi Omnicup, Clinical Innovations, Murray, UT, USA). 

Our general protocol entails careful monitoring throughout labor and after delivery, with timely documentation of either spontaneous bladder emptying or catheterization. During labor, absence of spontaneous micturition, within 4 hours, warrants intermittent catheterization. After two intermittent catheterizations, an indwelling catheter is positioned. In case of instrumental delivery or repair of severe genital tears requiring anesthesia or cesarean section, an indwelling catheter is left in situ for 12 h, and micturition is monitored after its removal. In case of PPUR or if PVRV is between 150 and 500 mL, clean intermittent catheterization every 4–6 h is ordered. If PVRV is over >500 mL, a permanent catheter is placed for 24 h and then removed, and patients are instructed to void spontaneously and thereafter assessed by ultrasonography. We adopted conventional definitions: Overt PPUR denotes inability to void six hours after vaginal delivery or catheter removal. Covert PPUR denotes the presence of urinary PVRV equal to or greater than 150 mL after spontaneous voiding, measured by ultrasound or catheterization. Persistent PPUR is defined by urinary retention requiring catheterization for more than three days. The UDI-6 questionnaire evaluates the presence and severity of urinary distress across three domains. The first domain (questions 1 and 2) addresses irritative symptoms, including frequency and urgency; the second domain (questions 3 and 4) concerns symptoms of urinary incontinence; and the third domain (questions 5 and 6) focuses on obstructive voiding symptoms.

Each item is scored on a four-point Likert scale: not at all (0); slightly (1); moderately (2); greatly (3). The sum, averaged and multiplied by 33.33 so as to obtain a score on a 0–100 scale, provides a total score that measures overall symptom distress [24]. A UDI-6 total score greater than 33.3 has previously been identified [24] as the threshold for greater distress from urinary symptoms. Accordingly, this cutoff was adopted in the present study.

The questions provided by the UDI-6 questionnaire are as follows:

Q1: How much are you bothered by frequent urination?

Q2: How much are you bothered by leakage related to feeling of urgency?

Q3: How much are you bothered by leakage related to activity, coughing or sneezing?

Q4: How much are you bothered by small amount of leakage (drops)?

Q5: How much are you bothered by difficulty emptying bladder?

Q6: How much are you bothered by pain or discomfort in lower abdominal or genital area?

### Statistical Analysis

The study was designed to compare asymptomatic women (*n* = 145) with those reporting lower urinary tract symptoms (LUTS) (*n* = 97). Using a significance level of 5%, the study had a statistical power of 97% to detect predictors with a medium effect size (Cohen’s d = 0.5 or h = 0.5). Descriptive statistics related to population characteristics, antenatal care, labor, birth variables, and fetal parameters were calculated. Categorical variables were summarized as frequency and percentage. Normally distributed continuous variables were reported as mean and standard deviation (SD). Skewed variables were reported as median and interquartile range. The chi-square test and Fisher’s exact tests were applied to compare categorical variables. An independent sample *t*-test and the Mann–Whitney test were used to compare continuous variables. Multivariable logistic regression was used to study the association between LUTS and variables that were significantly associated in the univariate analysis. All statistical tests were two-sided, and a *p*-value of less than 0.05 was considered statistically significant. SPSS software (IBM SPSS software for windows, version 28, IBM Corporation, Armonk, New York, NY, USA, 2021) was used for all statistical analyses.

## 3. Results

In the period of interest, an overall 2% baseline prevalence of PPUR was observed (*n* = 362/18,265). A total of 242 women (66.8%) completed the UDI-6 questionnaire, after a period of up to 10 years (median 6.4 years; IQR 4.7–8 years). Of these, 145 had no complaints, with a total score equaling zero (Group 1). Of the remaining 97 participants (Group 2), only 1 woman had a computed score greater than 33.33 considered indicative of high urinary distress, and 96 had minor urinary complaints with an overall score less than the 33.33 cutoff (mean 8.6 (±5.5); range 5.5–27.7). As such, significant long-term LUTS resulted in being negligible.

The baseline clinical characteristics are depicted in Table 1. The mean age in the studied population was 32.0 (±4.7) years (range 19 to 45). The rate of vacuum-assisted delivery was 16.94%. Known risk factors for PPUR, such as age, gestational week, parity, BMI, length of first and second stages of labor, vaginal tear, birth weight, head circumference, epidural analgesia, and a multitude of other items, were comparable between groups (Table 1). The only significant difference between groups was a preponderance of Caucasians in Group 2 (*p* = 0.012).

Dwell time was significantly longer in Group 1, but significance was overruled by multi-regression analysis (Table 2).

Within Group 2 (*n* = 97), irritative symptoms measured by questions 1 and 2 were reported by 23 participants. Incontinence measured by questions 3 and 4 was reported by 72 participants, and voiding difficulties provided by question 5 were reported by 15 women (15/242; 6.19%). Question no. 6 retrieved no positive answers. When scored on a four-point Likert scale, voiding difficulty was considered mild by most women (13/15; 86.7%) and moderate by two participants (2/15; 13.3%).

Participants with voiding difficulty (*n* = 15, item #5 ≥ 1) were compared to the rest of the cohorts (*n* = 227); epidural analgesia at delivery and primiparity resulted in being less frequent in the subgroup with voiding difficulty (*p* = 0.049 and *p* = 0.045, respectively), while uterine atony following birth and hypothyroidism (*p* = 0.047 and *p* = 0.045, respectively) were more frequent among the 15 participants with voiding complaints (Table 3). Caucasian ethnicity was not over-represented when participants with or without voiding difficulty were compared.

## 4. Discussion

Postpartum urinary retention is a relatively common condition that typically resolves following the use of a short-term urinary catheter, rarely proceeding beyond the 4th postpartum day. PPUR can remain underappreciated due to the low level of awareness amongst obstetricians. As PPUR may have, although rarely, upsetting long-term effects, early diagnosis and timely intervention may help to avoid long-term complications. Implementation of standardized voiding protocols helps to early identify, prevent, and efficiently treat PPUR. Prompt diagnosis and early active management, in the postpartum period, preclude lower urinary tract morbidity in the long run. Delays in diagnosis can occasionally contribute to bladder overdistension, leading to detrusor damage [22].

Our cross-sectional study investigated—using the UDI-6—the long-term effects of PPUR (median follow-up period of 6.42 years; IQR: 4.7–8, with a minimum of 3 years and up to 10 years follow-up) in a large cohort of women who delivered vaginally.

The rate of women (*n* = 362/18,265; 2%) who delivered vaginally and were granted the diagnosis of PPUR was low. A 2% prevalence rate is in line with the postulation that the incidence of PPUR in an institution with a suitable postpartum voiding protocol is low [6,14]. Two-thirds of parturients (*n* = 242/362) agreed to self-complete the UDI-6.

Using the largest cohort studied to date, our findings suggest that PPUR is generally associated with limited long-term sequelae, although a non-negligible minority of women did report persistent voiding symptoms. Early detection of PPUR and sound postpartum management therefore remain essential to minimize potential morbidity. The results of our investigation support previous observations that assert that PPUR has little or no lasting effects [12,19,20,21].

Although the vast majority of women report no clinically significant long-term symptoms, analysis of UDI-6 item 5 showed that 15 women in Group 2 (15/97 = 15.5%) experienced mild-to-moderate voiding difficulties. This finding underscores the need to refine risk stratification strategies so that women at higher risk of persistent or severe sequelae can be identified and managed earlier. Only one participant obtained a score that is indicative of severe urinary distress.

The rate of women with irritative symptoms (23/242; 9.5%) and incontinence symptoms (72/242; 29.8%) is comparable to that expected for young heathy women [25].

It has been previously shown that a multitude of confounders are independently and positively associated with PPUR [6]. These include age, advanced gestational age, nulliparity, premature rupture of membranes, labor induction, epidural analgesia, meconium-stained amniotic fluid, nonoperative vaginal birth, vacuum extraction, prolonged labor, episiotomy, perineal tear, and singleton pregnancy. Voiding before delivery was found to be preventive [18]. These well-established risk factors indicate that PPUR denotes a traumatic delivery-associated incident. Vacuum-assisted delivery ranks first in the list of events that predispose individuals to PPUR and is associated with a two days longer normalization interval [22]. It results that tough deliveries create momentary retention distress [8,17].

Participants from Group 1 (asymptomatic) vs. Group 2 did not differ on the multitude of confounders (Table 1) other than Caucasian ethnicity, which was more common in Group 2 (*p* = 0.012). Multi-regression analysis confirmed that Caucasian ethnicity was associated with long-term LUTS following PPUR. As such, Caucasians seem to be at increased risk for PPUR [6,26]. In order to further investigate the correlation between long-term voiding difficulties, years after enduring urinary retention following vaginal delivery, we processed a sub-analysis that compared women with long-term voiding distress (*n* = 15) and those free of voiding difficulties (*n* = 227). Uterine atony following birth and hypothyroidism were the only outcomes associated with long-term voiding difficulties (Table 3). Epidural analgesia at delivery and primiparity, considered major predictors of PPUR, resulted in being less frequent in the subgroup with voiding distress. The rate of epidural analgesia, known to be associated with greater residual urine after birth, and the rate of primiparity, known to be linked to prolonged and traumatic labor, were lower in women who displayed voiding distress years after delivery. This pattern supports the view that the transient component of PPUR is largely driven by peripartum factors, whereas long-term urinary retention likely reflects inherent pre-existing lower urinary tract vulnerabilities that are independent of childbirth.

Uterine atony diagnosed after delivery emerges as a predictor of long-term urinary retention. Otherwise, hypothyroidism emerges as a risk factor associated with long-term susceptibility to voiding difficulties probably unrelated to PPUR. Hypothyroidism is the only factor not related to birth confounders that was associated with long-term voiding difficulties in women who endured PPUR. This raises the question of whether hypothyroidism exposes parturients to PPUR and urinary retention thereafter. A significant correlation between TSH values and PVRV of more than 200 mL was demonstrated in an assembly of elderly women (mean age 84.4) with symptomatic urinary retention [27]. Otherwise, more than one-third of 725 women with LUTS were found to have hypothyroidism [28].

### Strengths and Limitations

To the best of our knowledge, this is the largest study—and one of the very few—specifically designed to evaluate the long-term sequelae of PPUR, and it includes the longest follow-up period reported. Its robustness derives from the high number of variables investigated (maternal characteristics, antenatal data, obstetric interventions, and birth- and neonatal-related factors) most of which showed no influence on long-term outcomes.

The analysis identifies hypothyroidism as an independent risk factor for voiding disfunction in women who endured PPUR, an association not previously highlighted on this scale. Moreover, the use of a validated, multilingual questionnaire will allow comparability in future studies. Finally, our findings reinforce current recommendations that early recognition and prompt, appropriate management of PPUR promote a rapid return to normal bladder function.

This study has several limitations, including its retrospective design, a moderate response rate (~67%), which may introduce non-response bias, and the absence of objective follow-up assessments using tools such as urodynamic testing. Although our study was powered to detect a medium effect size, we were able to identify even smaller effects (down to d = 0.37) with 80% power. However, in the subgroup analysis of women with voiding symptoms (*n* = 15), the study was only powered to detect large effect sizes (d = 0.75). Therefore, a further limitation is that within this subgroup, we cannot exclude the possibility that other predictors with smaller effect sizes exist. Future studies with larger sample sizes will be required to identify such associations.

## 5. Conclusions

PPUR is a relatively common self-limited condition that usually resolves within the first postpartum days. While most women remain asymptomatic in the long term, a clinically relevant subset reported ongoing voiding distress, particularly those with hypothyroidism or uterine atony. The results of this study sustain that PPUR is often a transient disturbance with minimal long-term consequences. Careful monitoring, early identification, enhanced postpartum surveillance, and knowledge of the risk factors are advocated.

## Figures and Tables

**Table 1:** Demographic and clinical characteristics. Data is presented as mean ± standard deviation, median (interquartile range), or N (%). *p* < 0.05 is considered significant.

	Group 1 Asymptomatic N = 145	Group 2 LUTS N = 97	*p*
**Maternal Characteristics**			
Age (years)	31.5 ± 4.6	31.8 ± 4.8	0.646
Caucasian ethnicity	118 (81.4%)	90 (92.8%)	0.012
Hypothyroidism	11 (7.6%)	11 (11.3%)	0.319
BMI (kg/m^2^)	22.35 ± 3.9	21.71 ± 3.1	0.365
Weight gain throughout pregnancy	13.35 ± 5.2	13.7 ± 4.8	0.83
**Pregnancy and Intrapartum Characteristics**			
Gestational age (weeks)	40.28 (39.0–41.0)	40.1 (38.8–0.7)	0.264
Nulliparity	125 (86.2%)	81 (83.5%)	0.631
GDM	20 (13.8%)	8 (8.2%)	0.186
PET/HTN	1 (0.7%)	1 (1%)	0.774
Thrombocytopenia	2 (1%)	1 (1%)	0.651
Preterm birth	5 (3.4%)	4 (4.1%)	>0.999
Induction of labor	53 (36.6%)	27 (27.8%)	0.354
Intrapartum antibiotic administration	25 (17.2%)	12 (12.4%)	0.302
Epidural anesthesia	94 (64.8%)	60 (61.9%)	0.638
Meconium-stained/hematic amniotic fluid	47 (32.4%)	25 (25.8%)	0.765
2nd stage of labor (mins)	85 (39–147)	75 (35–152)	0.915
Pushing phase (mins)	52 (30–86)	60 (30–90)	0.529
Prolonged second stage	31 (21.4%)	17 (17.5%)	0.461
Non-lithotomy birth position	22 (15.2%)	13 (13.4%)	0.701
Episiotomy	44 (30.3%)	37 (38.1%)	0.208
Vacuum-assisted delivery	25 (17.2%)	16 (16.5%)	0.879
**Postpartum Characteristics**			
Clinical estimation of blood loss (mL)	300 (200–500)	350 (225–500)	0.799
Hemoglobin drop (g/dL)	1.58 ± 1	1.66 ± 1.2	0.867
Perineal tear	81 (55.9%)	51 (52.6%)	0.615
OASI	2 (1.4%)	1 (1%)	0.957
Manual lysis of placenta	3 (2.1%)	1 (1%)	0.651
Postpartum uterine atony	3 (2.1%)	3 (3.1%)	0.686
Fever at postpartum	1 (0.7%)	4 (4.1%)	0.161
Persistent urinary retention (>72 h)	5 (3.4%)	6 (6.2%)	0.356
Duration of catheterization (days)	2 ± 4.5	1.88 ± 2.2	0.021
Interval between delivery and follow-up (years)	6.37 (4.62–8.1)	6.44 (4.73–7.73)	0.621
**Neonatal Characteristics**			
Birth weight	3312 ± 397	3330 ± 415	0.734
Macrosomia (>4 kg)	7 (4.8%)	4 (4.1%)	>0.999
SGA (<2.5 kg)	1 (0.7%)	0 (0%)	>0.999
Male	81 (55.9%)	51 (52.6%)	0.615
Newborn head circumference	34.5 ± 1.4	34.6 ± 1.3	0.382
Newborn height	50.1 ± 2	50.4 ± 2.1	0.138

Abbreviations: LUTS, lower urinary tract symptoms; BMI, body mass index calculated before or at the beginning of pregnancy; GDM, gestational diabetes mellitus; PET, preeclampsia; HTN, chronic or gestational hypertension; CVS, chorionic villus sampling; OASI, obstetric anal sphincter injury (there were no cases of 4th degree); SGA, small for gestational age defined as birth weight at term of less than 2500 g. The table is reprinted from the 16th EUGA Annual Congress Abstract book.

**Table 2:** Multivariable regression analysis for factors potentially associated with long-term LUTS.

	95% C. I	*p*
Age (years)	0.943–1.058	0.958
Time from birth (years)	0.828–1.102	0.531
Duration of catheterization (days)	0.916–1.044	0.503
Caucasian ethnicity	0.123–0.751	0.01

**Table 3:** Sub-analysis of women with or without voiding difficulties. Sub-analysis was based on response to question #5 of UDI-6. All parameters presented in Table 1 were analyzed and only those found to be statistically significant are presented in Table 3.

	Absence of Voiding Difficulties *n* =227	Voiding Difficulties *n* =15	*p*
Hypothyroidism	18 (7.9%)	4 (26.7%)	0.036
Epidural	148 (65.2%)	6 (40%)	0.049
Uterine Atony	4 (1.8%)	2 (13.3%)	0.047
Primiparity	199 (87.7%)	10 (66.6%)	0.045

## Data Availability

The data that support the findings of this study are not openly available due to reasons of sensitivity and are available from the corresponding author upon reasonable request.

## References

[1] Nutaitis A.C., Meckes N.A., Madsen A.M., Toal C.T., Menhaji K., Carter-Broks C.M., Propst K.A., Hickman L.C. (2023). Postpartum urinary retention: An expert review. Am. J. Obstet. Gynecol..

[2] Groutz A., Gordon D., Wolman I., Jaffa A., Kupferminc M.J., Lessing J.B. (2001). Persistent postpartum urinary retention in contemporary obstetric practice. Definition, prevalence and clinical implications. J. Reprod. Med..

[3] Yoshida A., Yoshida M., Kawajiri M., Takeishi Y., Nakamura Y., Yoshizawa T. (2022). Prevalence of urinary retention after vaginal delivery: A systematic review and meta-analysis. Int. Urogynecology J..

[4] Ain Q.U., Shetty N., Supriya K. (2021). Postpartum urinary retention and its associated obstetric risk factors among women undergoing vaginal delivery in tertiary care hospital. J. Gynecol. Obstet. Hum. Reprod..

[5] Pifarotti P., Gargasole C., Folcini C., Gattei U., Nieddu E., Sofi G., Buonaguidi A., Meschia M. (2014). Acute post-partum urinary retention: Analysis of risk factors, a case-control study. Arch. Gynecol. Obstet..

[6] Barba M., Frigerio M., Manodoro S., Bernascone D.P., Cola A., Palmieri S., Fumagalli S., Vergani P. (2021). Postpartum urinary retention: Absolute risk prediction model. Low. Urin. Tract Symptoms.

[7] Biurrun G.P., Gonzalez-Díaz E., Fernández C.F., Corona A.F. (2020). Post-Partum Urinary Retention and Related Risk Factors. Urology.

[8] Cao D., Rao L., Yuan J., Zhang D., Lu B. (2022). Prevalence and risk factors of overt postpartum urinary retention among primiparous women after vaginal delivery: A case-control study. BMC Pregnancy Childbirth.

[9] Buchanan J., Beckmann M. (2014). Postpartum voiding dysfunction: Identifying the risk factors. Aust. N. Z. J. Obstet. Gynaecol..

[10] Mulder F.E.M., Oude Rengerink K., van der Post J.A.M., Hakvoort R.A., Roovers J.-P.W.R. (2016). Delivery-related risk factors for covert postpartum urinary retention after vaginal delivery. Int. Urogynecology J..

[11] Mulder F.E.M., Schoffelmeer M.A., Hakvoort R.A., Limpens J., Mol B.W., van der Post J.A., Roovers J.P. (2012). Risk factors for postpartum urinary retention: A systematic review and meta-analysis. BJOG.

[12] Humburg J., Troeger C., Holzgreve W., Hoesli I. (2011). Risk factors in prolonged postpartum urinary retention: An analysis of six cases. Arch. Gynecol. Obstet..

[13] Liang G.C., Wong S.Y., Tsay P.T., Wong S.Y., Tsay P.T., Chang S.D., Tseng L.H., Wang M.F., Soong Y.K. (2002). The effect of epidural analgesia on postpartum urinary retention in women who deliver vaginally. Int. J. Obstet. Anesth..

[14] Hosakoppal S., Brown O., Peaceman A. (2022). Postpartum urinary retention after the institution of a universal voiding protocol. J. Matern. Fetal Neonatal Med..

[15] Groutz A., Levin I., Gold R., Pauzner D., Lessing J.B., Gordon D. (2011). Protracted postpartum urinary retention: The importance of early diagnosis and timely intervention. Neurourol. Urodyn..

[16] Dolezal P., Ostatnikova M., Balazovjechova B., Psenkova P., Zahumensky J. (2022). Covert postpartum urinary retention: Causes and consequences (PAREZ study). Int. Urogynecology J..

[17] Gupta A., Pampapati V., Khare C., Murugesan R., Nayak D., Keepanasseril A. (2021). Postpartum urinary retention in women undergoing instrumental delivery: A cross-sectional analytical study. Acta Obstet. Gynecol. Scand..

[18] Lamblin G., Chene G., Aeberli C., Soare R., Moret S., Bouvet L., Doret-Dion M. (2019). Identification of risk factors for postpartum urinary retention following vaginal deliveries: A retrospective case-control study. Eur. J. Obstet. Gynecol. Reprod. Biol..

[19] Mulder F.E.M., Hakvoort R.A., de Bruin J.P., Janszen E.W., van der Post J.A.M., Roovers J.W.R. (2018). Long-term micturition problems of asymptomatic postpartum urinary retention: A prospective case-control study. Int. Urogynecology J..

[20] Zussman N.M., Gonen N., Kovo M., Miremberg H., Bar J., Condrea A., Ginath S. (2014). Protracted postpartum urinary retention-a long-term problem or a transient condition?. Female Pelvic Med. Reconstr. Surg..

[21] Mulder F.E.M., Hakvoort R.A., Schoffelmeer M.A., Limpens J., van der Post J.A.M., Roovers J.W.R. (2014). Postpartum urinary retention: A systematic review of adverse effects and management. Int. Urogynecology J..

[22] Mohr S., Raio L., Gobrecht-Keller U., Imboden S., Mueller M.D., Kuhn A. (2022). Postpartum urinary retention: What are the sequelae? A long-term study and review of the literature. Int. Urogynecology J..

[23] Barba M., Cola A., Melocchi T., Cola A., Melocchi T., Braga A., Castronovo F., Manodoro S., Pennacchio M., Munno G.M. (2023). Italian validation of the Pelvic Floor Distress Inventory (PFDI-20) questionnaire. Int. Urogynecology J..

[24] Skorupska K., Grzybowska M.E., Kubik-Komar A., Rechberger T., Miotla P. (2021). Identification of the Urogenital Distress Inventory-6 and the Incontinence Impact Questionnaire-7 cutoff scores in urinary incontinent women. Health Qual. Life Outcomes.

[25] Zilberlicht A., Boms-Yonai N., Haya N., Feferkorn I., Lavie O., Abramov Y. (2020). Somatic and psychological triggers for bladder storage symptoms among men and women. Int. Urogynecology J..

[26] Teo R., Punter J., Abrams K., Mayne C., Tincello D. (2007). Clinically overt postpartum urinary retention after vaginal delivery: A retrospective case-control study. Int. Urogynecology J..

[27] Justo D., Schwartz N., Dvorkin E., Gringauz I., Groutz A. (2017). Asymptomatic urinary retention in elderly women upon admission to the Internal Medicine department: A prospective study. Neurourol. Urodyn..

[28] Zargham M., Hajian M.R., Alizadeh F., Eslami M.J., Boroujeni N.K., Gholipour F. (2022). Hypothyroidism is prevalent among adult women with chronic lower urinary tract symptoms. Low. Urin. Tract Symptoms.

